# Cell Senescence and Central Regulators of Immune Response

**DOI:** 10.3390/ijms23084109

**Published:** 2022-04-07

**Authors:** Sergey M. Lunin, Elena G. Novoselova, Olga V. Glushkova, Svetlana B. Parfenyuk, Tatyana V. Novoselova, Maxim O. Khrenov

**Affiliations:** Institute of Cell Biophysics, Russian Academy of Sciences, PSCBR RAS, Institutskaya Str. 3, 142290 Moscow, Russia; elenanov_06@mail.ru (E.G.N.); glushckova@mail.ru (O.V.G.); lana_kras2@rambler.ru (S.B.P.); novossulova_t@rambler.ru (T.V.N.); xpehob2004@mail.ru (M.O.K.)

**Keywords:** cell senescence, cell cycle, immune response, neuroendocrine regulators, hypothalamic–pituitary–adrenal axis, neurotransmitters, thymic hormones, melatonin, adrenaline, acetylcholine, thymosines

## Abstract

Pathways regulating cell senescence and cell cycle underlie many processes associated with ageing and age-related pathologies, and they also mediate cellular responses to exposure to stressors. Meanwhile, there are central mechanisms of the regulation of stress responses that induce/enhance or weaken the response of the whole organism, such as hormones of the hypothalamic–pituitary–adrenal (HPA) axis, sympathetic and parasympathetic systems, thymic hormones, and the pineal hormone melatonin. Although there are many analyses considering relationships between the HPA axis and organism ageing, we found no systematic analyses of relationships between the neuroendocrine regulators of stress and inflammation and intracellular mechanisms controlling cell cycle, senescence, and apoptosis. Here, we provide a review of the effects of neuroendocrine regulators on these mechanisms. Our analysis allowed us to postulate a multilevel system of central regulators involving neurotransmitters, glucocorticoids, melatonin, and the thymic hormones. This system finely regulates the cell cycle and metabolic/catabolic processes depending on the level of systemic stress, stage of stress response, and energy capabilities of the body, shifting the balance between cell cycle progression, cell cycle stopping, senescence, and apoptosis. These processes and levels of regulation should be considered when studying the mechanisms of ageing and the proliferation on the level of the whole organism.

## 1. Introduction

The ageing of an organism is based on the mechanisms that mediate the ageing of its cells, that is, cell senescence. Senescence is a programmed mechanism that protects cells, predominantly against DNA damage. Senescent cells stop dividing in response to mitogenic stimuli, and this condition is irreversible. In addition, such cells acquire certain morphological and functional changes that distinguish them from both the dividing and the resting (G_0_ phase) cells. Senescent cells partly retain their tissue-specific functions and therefore are not completely lost from the body. Senescence, from both a mechanical (signalling pathways) and a functional point of view, is parallel and complementary to apoptosis in many parameters; apoptosis is another cellular response mechanism to damage. Both apoptosis and senescence are involved in embryonic development and regeneration. Both these mechanisms control cell proliferation and proliferative diseases, including cancer, and are associated with disturbances in these processes. From an evolutionary point of view, these mechanisms are ancient and are present in all multicellular organisms [1].

The transition of a cell to a senescent state or to death through apoptosis may be induced by either internal factors (DNA damage, oxidative stress, disturbances of metabolic systems) or external ‘commands’ that are transmitted mainly through receptors and affect the internal balance of signalling pathways. External signals may be derived from the immediate environment of cells, mainly through paracrine networks of signalling molecules, such as cytokines, chemokines, and growth factors. These molecules are produced, for example, by immune cells as a part of their functions, or senescent cells, as a part of the senescence-associated secretory phenotype (SASP). Such signals may force the surrounding cells into senescence and, in turn, promote local inflammation and attract cells of the immune system [2]. 

Additionally, there are endocrine systems. It is known that the body is influenced by internal rhythms, diurnal and annual, which are tied to periodic factors such as light and temperature changes, and also react systemically to stressors affecting the body as a whole. All of these functions are mediated through internal hormonal systems, which undoubtedly affect the cell cycle and, accordingly, the mechanisms underlying cell senescence and proliferation. 

Although there have been several studies on the hypothalamic–pituitary–adrenal (HPA) axis and whole organism ageing [3], we found no study that systematically analysed the relationship between the neuroendocrine regulators of stress and inflammation and intracellular mechanisms controlling cell cycle, senescence, and apoptosis. This review was aimed at filling this gap. We investigated the available data concerning the effects of the HPA axis and the mediators of the parasympathetic and sympathetic systems mediating responses to stressful influences, several thymus hormones that specifically affect the immune cells and are often considered as belonging to the stress-response system, and the hormone melatonin, a primary endocrine regulator of circadian rhythms, on signalling cascades associated with the control of cell cycle, senescence, and apoptosis.

The next two sections provide a brief description of cell senescence. 

## 2. Causes of Cell Senescence

One of the basic causes of senescence is the finite number of divisions initially programmed in cells [4], called Hayflick’s limit. After this number of divisions, the main population of cells in the human body enters an irreversible state of cell cycle arrest called replicative senescence. The underlying mechanism for this is associated with the telomere, that is, the repeating DNA sequence TTAGGG present at the ends of linear chromosomes, protecting the chromosomes from degradation and recombination. Telomeres shorten with each cell cycle due to features of the DNA replication process, such as RNA priming of the lagging strand and the unidirectional action of DNA polymerases. Thus, telomere length is a factor that limits the number of cell divisions [5]. In stem cells and many cancer cells, the enzyme telomerase is expressed; it adds DNA repeats to telomeres, and consequently, cells acquire the ability to divide indefinitely. However, telomerase is not expressed in most cells, and as soon as telomeres become too short, a DNA damage response (DDR) is initiated, leading to the activation of cell cycle inhibitors and resulting in cell senescence [6]. Thus, with age, the body gradually accumulates an increasing number of senescent cells.

However, telomere shortening is not the only cause of senescence. Senescence can also be induced by proliferative stressors such as the over-activation of oncogenes such as Ras [7] and BRAF [8] and the dysfunction of tumour suppressors [9]. Therefore, senescence is a natural defence mechanism against tumours.

Moreover, many factors of an aggressive and stressful nature, particularly reactive oxygen species (ROS), X-rays, ultraviolet radiation, and DNA-damaging agents used in cancer chemotherapy, are capable of initiating DDR and, consequently, senescence. All these factors may lead to DNA damage and induction of senescence through DDR-dependent mechanisms. Senescence may also be activated under conditions of inflammation associated with the excessive release of aggressive compounds by immune cells, such as ROS.

Senescence induces noticeable morphological changes in cells, such as increased volume, smoother shape, and increased intercellular spaces. Cells also demonstrate some features, such as senescence-associated beta-galactosidase (SA-β-Gal) activity, that are usually not observed in normal cells and therefore are used as markers of senescence. Additionally, DNA synthesis is blocked in senescent cells, the foci of DNA damage are visible, satellite DNA appears bloated, and increased expression of some microRNAs is observed. In addition, senescent cells secrete many factors such as cytokines, growth factors, matrix remodelling proteins, proteases, and chemokines, which are collectively referred to as the senescence-associated secretory phenotype (SASP) [10].

The most prominent component of SASP is IL-6, a pleiotropic pro-inflammatory cytokine whose secretion is increased by DDR-dependent and oncogene-induced senescence in a variety of cell types [11,12,13,14]. In senescent cells, increased expressions of IL-1 and IL-8 were observed, and the expressions of IL-6 and IL-8 were upregulated through IL-1 [15]. Most senescent cells also express chemokines, for instance, those belonging to the CCL family [15]. Senescent epithelial cells, endothelial cells, and fibroblasts express high levels of almost all proteins that bind insulin-like growth factors, including IGFBP-2, -3, -4, -5, and -6 [13,16,17], and their regulators, IGFBP-rP1 and IGFBP-rP2 [18,19]. SASP also includes a number of matrix metalloproteases (MMPs), such as MMP-1, -3, and -10 [20,21,22,23,24], and serine proteases and their endogenous inhibitors [25]. In addition, senescent cells release nitric oxide and ROS due to changes in the levels of nitric oxide synthase and superoxide dismutase activity [26,27,28,29,30]. There is evidence that the generation of SASP in senescent cells is associated with the activation of the NF-κB signalling pathway [1], a primary regulator of the proinflammatory response in cells.

Cellular senescence is physiologically normal during embryonic development and in adult organisms. In embryonic tissues, the SASP of senescent cells includes signalling molecules that stimulate morphogenesis. In adult tissues, SASP plays an important role in wound healing, as it suppresses carcinogenesis and stimulates regeneration; however, the underlying mechanisms are different from those in embryonic tissues [2].

Therefore, SASP has a protective function, because it attracts cells of the immune system designed to eliminate damaged and senescent cells and also contributes to the normal processes of inflammation, wound healing, tissue remodelling, and cellular plasticity [31,32,33,34]. On the other hand, these factors can disrupt tissue homeostasis, contributing to the senescence of surrounding cells (paracrine ageing) [35], and chronic inflammation may lead to pathological conditions such as fibrosis and cancer [32]. Long-term exposure to SASP factors may lead to the development of an inflammatory disorder, since it includes a number of pro-inflammatory mediators. As noted above, inflammation is associated with the recruitment of immune cells, the release of aggressive mediators, and stress exposure of the surrounding cells. Long-term chronic exposure to inflammation factors formed during both ‘normal’ chronic inflammation and the accumulation of a large number of senescent cells on the surrounding intact cells may result in stress activation of signalling pathways associated with the arrest of the cell cycle and the initiation of senescence mechanisms.

## 3. Cell Cycle Arrest as a Response to Stress Exposure

**Figure 1:**
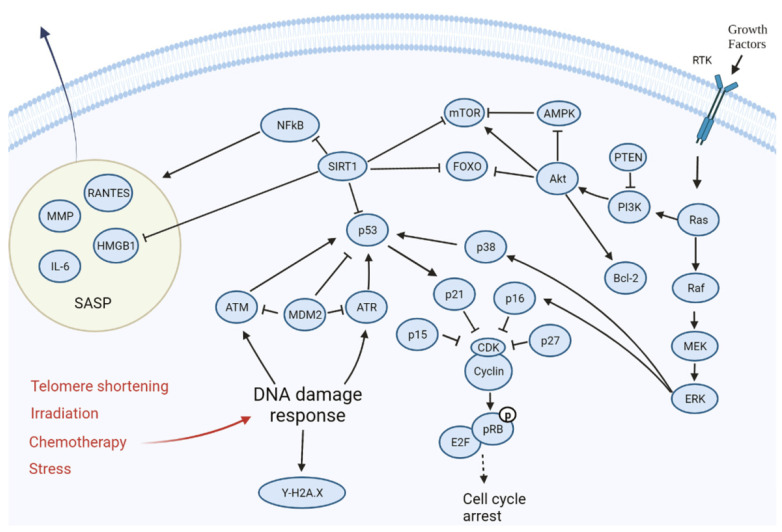
Simplified representation of signalling pathways related to cell senescence.

Cell cycle arrest is one of the early responses of cells to stress exposure, and it is mainly realised through temporary or permanent inhibition of cyclin-dependent kinases (CDC). Many studies indicate that the fate of a cell is determined by the stress level, and the strength and nature of the stress determines whether the cell will re-enter the normal cell cycle, enter an irreversible state of senescence, or immediately die through apoptosis. Indeed, many studies have shown that apoptosis is a response to excessive stress, whereas senescence is a response to less severe damage. In particular, it has been shown that the DNA-damaging agent doxorubicin at low doses causes senescence, and at higher doses, apoptosis [36]. The same dose dependence was observed with other damaging agents, such as etoposide [37], UV radiation [38], and oxidative stress induced by the addition of hydrogen peroxide [39,40]. The primary mechanisms underlying this dependence and determining the fate of the cell are a network of signalling pathways including the PI3K-AKT-mTOR, Ras-MEK-ERK, p53-p21-Rb, and p16-Rb pathways. A simplified scheme of these pathways is presented in Figure 1. The relationships within and between these systems are very complex and have been investigated in several publications. For instance, excellent reviews on these relationships are presented in [1,10] and [41]. 

In particular, a low level of stress and, consequently, a low p16 level, leads to a temporary arrest of the cell cycle, while a higher level of stress leads to senescence. Low and temporary increases in p53 expression and acetylation of p53 at Lys 161/Lys162 contribute to cell cycle arrest and senescence, while higher levels of p53, acetylation at Lys117, and interaction of DNA-binding domains within p53 lead to the transcription of proapoptotic genes and induction of apoptosis [1].

There has been evidence that the temporary or permanent nature of cell cycle arrest may depend on the activity of the PI3K/Akt/mTOR signalling pathway [42,43]. The mammalian target of rapamycin (mTOR) is a catalytic subunit of two protein complexes, mTORC1 and mTORC2. These complexes regulate cell growth and proliferation [44,45,46]. mTOR is inactive in cells with a temporary arrest of the cell cycle, whereas it remains active in senescent cells [42]. Because the cell cycle in senescent cells is arrested, they are not susceptible to the pro-proliferative action of the PI3K/Akt/mTOR cascade but respond to their growth-causing effects; therefore, senescent cells continue to increase in size and become larger than usual [47]. Senescence-related hypertrophy also leads to an increase in the number and size of lysosomes, which is associated with the activity of lysosomal β-galactosidase (SA-β-gal), an important feature used to identify senescent cells [48].

Furthermore, a distinction should be made between the normal physiological role of senescence in the development of tissues and organs, in responses to stress, and in regenerative processes and the pathological accumulation of senescent cells. The pathological accumulation of senescent cells may be associated with a reduced ability to stabilise the p53 protein due to the senescence-related modifications to p53 (which lead to impaired activation of apoptotic mechanisms) and with the release of cells from the immune surveillance [1]. In this regard, the question of how to increase the sensitivity of the immune system to SASP components so that it can eliminate accumulating senescent cells and how to modulate inhibited proapoptotic pathways in such cells seems to be very relevant. 

Considering that ageing-related pathologies, such as osteoarthritis, are based on cellular senescence mechanisms, variety of anti-senescence agents have been discovered and developed. These agents may be divided into several categories: sirtuin (SIRT)-activating drugs, senolytics, and senomorphics. These agents take their effect either by inducing apoptosis of senescent cells and or suppressing harmful activity of the SASP factors [49]. For example, cyanidin, a SIRT6 activator, significantly decreases IL-1β-induced expression of TNF-α, IL-6, COX-2, MMP-13, and ADAMTS-5, thereby preventing synovial inflammation and cartilage degradation in mice with osteoarthritis [50]. Fisetin, flavonoid senolythic, may take effect through PI3K/AKT/mTOR and NF-κB pathways and significantly downregulates the expression of p16INK4A and effectively removes senescent cells in premature ageing mice. It also prolongs the healthy lifespan of aged mice and reduces age-related pathological changes [51]. Rapamycin is a potent senomorphic and SASP inhibitor, although it is unable to reverse cellular senescence. It inhibits the expression of IL-1 by suppressing mTORC1 activity, while the reduction of IL-1α blocks the production of other SASP factors including IL-6, IL-8, CSF-2, CCL-8, and BMP-4 through inhibition of NF-κB pathway, thus, alleviating the senescence-related inflammation [52].

New perspectives for solving such problems may be obtained by studying the role of central neuroendocrine mechanisms in the regulation of senescence. In addition, a decrease in the level of chronic inflammatory responses in tissues may promote the restoration of normal physiological mechanisms. Of particular interest is the central regulation of the stress response in the body, the HPA, whose hormones are directly involved in regulating inflammation.

## 4. Neuroendocrine Regulators of Immune Response and Cell Senescence

We divided the remaining part of the review into subsections, dedicated to primary neuroendocrine factors that can directly contact with peripheral tissues and influence them: glucocorticoids, neurotransmitters of sympathetic and parasympathetic systems, thymic hormones, and melatonin. In each subsection, we describe accumulated data on their effects on intracellular pathways related to cell cycle regulation and provide a brief summary.

The central part of the stress response system is located in the hypothalamus and the brain stem. In the hypothalamus, the stress response system utilizes two groups of neurones of the paraventricular nucleus: those that secrete corticotropin-releasing hormone (corticoliberin) and those that secrete arginine-vasopressin. The stress response system also involves the corticoliberin neurones of the paragigantocellular and parabranchial nuclei of the medulla oblongata, noradrenergic locus coeruleus, and other predominantly noradrenergic groups of neurones in the medulla oblongata, spinal cord, and pons, which are part of the adrenergic sympathetic system [53].

The peripheral part of the stress response system is the hypothalamic–pituitary–adrenal axis and the components of the efferent part of the sympathetic and the parasympathetic systems.

Corticoliberin is the primary hypothalamic regulator of the HPA system. It plays a decisive role in the development of the stress response. Corticoliberin and arginine-vasopressin are secreted by hypothalamic neurones, leading to the production of adrenocorticotropic hormone (ACTH) by pituitary cells. Normally, the concentration of corticoliberin and arginine-vasopressin in the pituitary portal system fluctuates according to the circadian (daily) rhythm, which is influenced by factors such as light exposure, diet, and physical activity. When a stressor is presented, this rhythm is disturbed, the amplitude of the pulsations increases sharply, and a complex of system-wide reactions involving the sympathetic and the parasympathetic systems is triggered. The main target of ACTH is the adrenal cortex, which is a regulator of glucocorticoid secretion; glucocorticoids play a key role in inhibiting the stress response. Activation of the HPA axis has a distinct inhibitory effect on the immune response, because almost all components of the immune response are suppressed by the adrenal glucocorticoid hormone cortisol [54,55]. Thus, stimulation of the HPA axis triggers a negative feedback loop that inhibits the stress response.

In particular, glucocorticoids affect the functions and motility of leukocytes, suppress the production of inflammatory mediators and cytokines, and reduce their effects on tissues. These effects are observed both during the inflammatory process and under normal conditions, depending on circadian changes in the concentration of glucocorticoids in the blood. There is evidence showing circadian variations in glucocorticoid levels in some immune functions [55].

In the following sections, the available data on the effects of the regulators of immune responses on cell senescence- and cell cycle control-associated processes are explored.

### 4.1. Glucocorticoids

The effects of glucocorticoids on senescent cells and the induction of senescence have been studied previously to some extent. In particular, administering cortisol, the main glucocorticoid produced by the human adrenal glands, and corticosterone, the main rodent glucocorticoid, was shown to reduce the production of some SASP components, such as IL-6, in human fibroblasts during senescence induced by radiation or mitogenic stimuli. In addition to IL-6, the secretion of IL-8, GM-CSF, and MCP-2 also decreased, but none of the glucocorticoids reduced the levels of all SASP components. Cortisol acts through the cytoplasmic glucocorticoid receptor, which, by binding to its ligand, inhibits IL-1α signalling and transactivation of the NF-κB signalling pathway [56].

It was also shown that under conditions of chemically induced acute renal failure with oxidative stress, cortisol led to a significant increase in the level of the p21 protein, an important marker of activation of the pathways leading to cell cycle arrest and senescence in kidney cells. This increase was blocked by a corticosterone inhibitor but not a p53 inhibitor; that is, the increase was p53-independent. Moreover, cortisol and dexamethasone induced an increase in p21 levels in normal mice without inducing acute renal failure [57].

Another study showed the induction of senescence in human tenocytes using dexamethasone. Cells express senescence markers such as SA-β-gal. This study showed the activation of p21 expression and mTOR signalling in these cells, indicating the irreversible nature of cell cycle arrest. Simultaneously, the activation of p53 and the inhibition of sirtuin-1, a deacetylase of the p53 protein, were shown. Knockdown or inhibition of p53 was shown to prevent senescence [58]. In addition, the glucocorticoid receptor (GR) agonist dexamethasone reversibly arrested the cell cycle of tumour cells, with increased expression of the glucocorticoid receptor in the G_1_ phase, and following the withdrawal of dexamethasone, the cells slowly resumed the cycle [59]. Simultaneously, the same authors showed that long-term exposure to dexamethasone led to temporary p21 activation in lung adenocarcinoma cells with increased GR expression, which led to an irreversible arrest of clonal growth associated with p27^kip^ activation; the cells acquired a phenotype associated with senescence, depending on the activation of p27^kip^. The irreversible cycle arrest depended on the level of GR expression in cells and the dose of dexamethasone. This effect was independent of p21 activation and did not lead to p16 activation. In addition, the effect was independent of known factors involved in p27^kip^ regulation, such as FOXO3/FOXO4 [60]. Dexamethasone also induced p21-dependent senescence in human pluripotent placental epithelial cells with shortening of telomeres but without an increase in the number of replications [61]. The authors suggested that this effect may be related to telomerase activity.

Thus, glucocorticoids cause temporary (quiescence) or permanent (senescence) cell cycle arrest.

There have also been studies that show a seemingly opposite effect. For instance, glucocorticoids reduce hyperactive B-RAF kinase-induced senescence in human fibroblasts by reducing the transcription of the p21 and p15 proteins involved in senescence induction [62]. However, it should be noted that this senescence variant did not seem to be associated with DDR and p53 activation. Additionally, p21 and p15 activation occurred through the growth-factor-activated BRAF-MEK-ERK pathway, which may act as a hyperproliferation sensor, rather than through the classical DDR pathway via p53. Moreover, sustained activation of the proliferation-associated BRAF-MEK-ERK pathway has been observed in cancer cells in which cell cycle regulation is altered, and this should be taken into account when considering the effects of hormones.

Glucocorticoids may be effective inducers of apoptosis [63,64,65]. The relationship between the pro-apoptotic response to glucocorticoids and the functionality of p16 was shown in a lymphoblastic B-cell line, in which blocking p16 expression using siRNA led to a decrease in glucocorticoid-induced apoptosis [66]. The pro-apoptotic response is a protective mechanism for systemically inhibiting the immune response and preventing pathological activation. This effect of glucocorticoids is also widely used in the clinic for treating lymphoproliferative diseases [67].

However, in the case of some tumours, such as epithelial tumours, glucocorticoids provide an anti-apoptotic, cytoprotective effect, which is clinically unfavourable, because it makes tumour cells more resistant to cytotoxic drugs. It is known that many tumour cell lines have an increased expression of the glucocorticoid receptor [68]. As noted above, the cell cycle was temporarily arrested with the expression of senescence markers when exposed to corticosteroids [59,60]; this probably serves as a defence mechanism against apoptosis induction and leads to drug resistance [69]. Another type of cell in which glucocorticoids induce apoptosis is osteoblasts, and p21 blockade enhances this effect [70]. The apoptotic effect on osteoblasts is associated with the long-term use of glucocorticoids against various diseases and is obviously an adverse side effect as it leads to osteoporosis.

**Figure 2:**
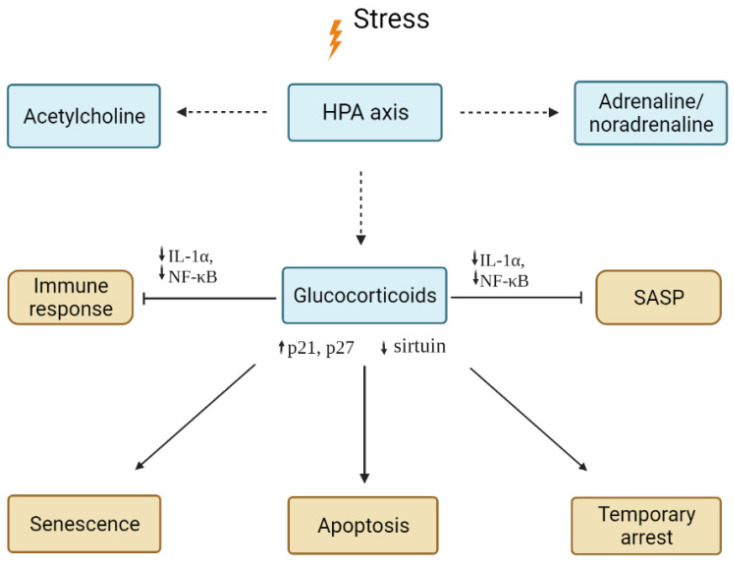
Schematic representation of effects of glucocorticoids on intracellular processes and signalling pathways related to senescence, apoptosis, and cell cycle regulation. ↑—stimulation, ↓—inhibition.

*Summary:* It can be concluded (Figure 2) that glucocorticoids secreted by the adrenal glands have a well-known and pronounced anti-inflammatory effect on all components of the immune response, besides reducing the production of a number of pro-inflammatory compounds secreted by senescent cells. In addition, they can activate the processes of cell cycle arrest, senescence, or apoptosis in cells exposed to acute stress, acting mainly through mechanisms associated with DDR. Response to glucocorticoids, apoptosis, senescence, or simply a temporary arrest of the cell cycle may vary depending on the cell type and specific conditions.

Thus, the systemic effect of glucocorticoids in the body is aimed at inhibiting the inflammatory response and temporarily or permanently stopping the division of cells exposed to stress. However, it is clear that prolonged stress associated with high levels of corticosteroids may lead to adverse effects, such as degenerative effects associated with cell cycle arrest. There are also conditions in which, as a result of chronic social stress, there is a parallel rise in the levels of glucocorticoids and pro-inflammatory cytokines [71]. Such an increase indicates a disruption of the feedback in the stress response system and inhibits the healing process [72,73], which may lead to pathologies [74,75]. Indeed, it has been demonstrated that a chronic increase in glucocorticoid levels accelerated cellular ageing and increased disease susceptibility in various species, including humans [76], monkeys [77], and rats [78].

### 4.2. Neurotransmitters of the Sympathetic and the Parasympathetic Systems

The autonomic nervous system consists of the sympathetic and the parasympathetic components and is also an efferent part of the stress response system. The main mediators of the autonomic nervous system include adrenaline, noradrenaline, and acetylcholine, which are released by nerve endings, smooth muscle cells, and vascular epithelium; these are capable of regulating the activity of the immune system and influencing the activation and inhibition of pathways associated with cell cycle arrest and senescence.

The central activation of stress by the autonomic nervous system leads to the activation of the immune system, which is mediated mainly through sympathetic fibres innervating the lymphoid organs, which transmit adrenergic signals to the α-adrenergic receptors of leukocytes located in these organs, inducing immune responses, such as the release of cytokines [79,80]. In contrast, the parasympathetic nervous system inhibits the activity of the immune system and inflammation [81,82], thus completing the so-called immune reflex. This effect was shown to be mediated via cholinergic signalling through the α7 subunit of the nicotinic acetylcholine receptor on macrophages, and it causes a decrease in the production of TNF-α [83,84] IL-1β, IL-6, and IL-8 [85] and a decrease in neutrophil recruitment and secretion of pro-inflammatory mediators [86,87].

Neurotransmitters of the sympathetic and the parasympathetic systems may accumulate in tissues in significant amounts, both as a result of the activity of these systems and due to production from tissues. In addition, cells of various tissues have receptors for these transmitters, which induce various effects in these tissues. It should be noted that the receptors influence a number of overlapping signalling pathways, such as adenylate cyclase (AC)/protein kinase A (PKA) and phospholipase C (PLC)/protein kinase C (PKC), and they are closely associated with changes in the intracellular concentrations of messengers such as cAMP and intracellular Ca. Almost all of these receptors are G-protein-coupled receptors that affect calcium depots, except for nicotinic acetylcholine receptors, which are ion channels for the direct entry of calcium and sodium ions. Thus, the neurotransmitters of the sympathetic and the parasympathetic systems play an important role in the regulation of the internal state of cells; their role in the regulation of processes associated with cellular ageing and the DDR response seems to be of great interest.

#### 4.2.1. Adrenalin and Noradrenalin

Adrenergic stimulation induces effects in various tissues that are aimed at implementing the ‘fight or flight’ strategy, and this influences the activities of metabolic signalling pathways. In adipose tissue, adrenergic stimulation induces lipolysis to generate high-energy substrates for peripheral tissues (white adipose tissue) or thermogenesis (brown adipose tissue). In the skeletal muscle, the effects are hypertrophy and modulation of glucose uptake, and in smooth muscle, the effect is relaxation. Many studies have shown that these effects of adrenergic stimulation are mediated via the mTOR kinase signalling pathway, which is one of the most important regulators of anabolism/catabolism and protein synthesis. The pathway includes two protein complexes: mTORC1 that activates protein synthesis [88,89], lipid synthesis [90,91], and mitochondrial formation [92] and inhibits autophagy [93], while mTORC2 regulates cellular proliferation via the Akt pathway [94] and rearrangement of the cytoskeleton via PKC signalling [95]. Adrenoreceptors may induce the activation of the mTORC1 complex through translocation of an inhibiting protein, REDD1, and may also activate the mTORC2 complex, thereby modulating the glucose uptake in adipose tissue and skeletal muscles [96].

Activation of the β-adrenergic receptor leads to a significant increase in cAMP levels in the cytoplasm. This increase is sudden and reaches a high level in minutes. However, with continued stimulation of the receptor by an agonist, the cAMP level dropped to its original value due to receptor desensitisation [97,98]. When the receptor is desensitised, it is phosphorylated and bound to arrestin proteins, which are adapters for nonclassical signalling pathways. Activation of the β-arrestin1 protein by adrenergic receptor β2AR activated the ubiquitin ligase MDM2 through the AKT signalling pathway [99]. In addition, the AKT signalling pathway was directly activated by PKA. This activation resulted in the binding of MDM2 to p53 and degradation of p53 [100]. Moreover, signalling through beta2-adrenergic receptors led to the dephosphorylation of β-arrestin2 and suppression of the NF-κB signalling pathway, an important activator of the DDR response. Such activation disrupts the operation of DDR systems, which ultimately leads to the accumulation of phosphorylated γ-H2AX histones inside the nuclei [100]. In another study by Hara et al. [101], the pharmacological blockade of this β-arrestin1-mediated effect on p53-MDM2 effectively reduced behavioural stress-induced DNA damage.

It has also been shown that physiological concentrations of noradrenalin and adrenalin rapidly induce DNA damage and disrupt repair processes in pre-cancerous cells (by acting on Chk1 and Chk2 kinases), causing the transformation of such cells into cancer cells [102,103]. In cancer cells, noradrenalin causes cell cycle arrest with increased p21 expression and γH2AX phosphorylation. The mechanism of this effect includes the activation of ATR kinase, which responds to DNA single-strand breaks (unlike another kinase, ATM, which responds to double-strand breaks), and subsequent activation of Chk1 kinase and its target CDC25, leading to cell cycle arrest. The authors noted that another stress hormone, cortisol, showed a similar effect [104].

In addition, long-acting β2-adrenergic receptor agonists led to an increase in the expression of p21^Cip^ and p27^Kip^ in smooth muscle cells in the human airway through a cAMP-dependent mechanism; however, this increase did not lead to a permanent arrest of the cell cycle, as the use of the growth factor PDGF-BB activated proliferation [105].

In some tissues (e.g., cardiac tissue), beta-adrenergic stimulation induced activation of the Raf/MEK/ERK cascade, leading to hypertrophy, that is, a pro-proliferative effect [106]. It should be noted that this opposing effect relative to the DDR-associated cascades is somewhat consistent with the aforementioned effect of glucocorticoids, which induced cell cycle arrest and senescence in response to DNA damage, but under conditions of stable activation of the Raf/MEK/ERK cascade decreased senescence [62]. In the prostate cancer cell line PC-3, catecholamines showed a pro-proliferative effect and the α1-adrenergic receptor inhibitor abedipinedilol-A showed an antiproliferative effect, causing cell cycle arrest, an increase in p21 and p27 expression, and an increase in apoptosis [107]. However, it should be noted that in these cells, the oncogenic mutation is associated with PTEN. Activation of the Raf/MEK/ERK cascade, on the contrary, is reduced and, perhaps as a result, p21 activation is suppressed, which allows these cells to evade cell cycle arrest [108]. Consequently, signalling associated with cell cycle regulation and senescence is usually altered in cancer cells; therefore, as noted above, the effects of hormones and mediators on these cells should be considered with caution.

Moreover, G-protein-independent activation of the ERK pathway has been shown with β-adrenergic receptors [109].

The highly selective α2-adrenergic agonist dexmedetomidine reduced renal ischemia-reperfusion-induced acute kidney injury (AKI) and subsequent renal fibrosis. Simultaneously, the expression of p53, p21, and p16 decreased. In addition, the levels of pro-inflammatory markers in kidneys with AKI decreased. The action of this agonist was similar to that of rapamycin, an mTOR inhibitor [110]. The protective effect of dexmedetomidine has also been shown in liver cells under ischaemia-reperfusion conditions, and it was realised by suppressing the activity of the NF-κB signalling cascade [111]. These results are consistent with the findings that the effects of α2-adrenergic receptors are often opposite to those of β-adrenergic receptors.

**Figure 3:**
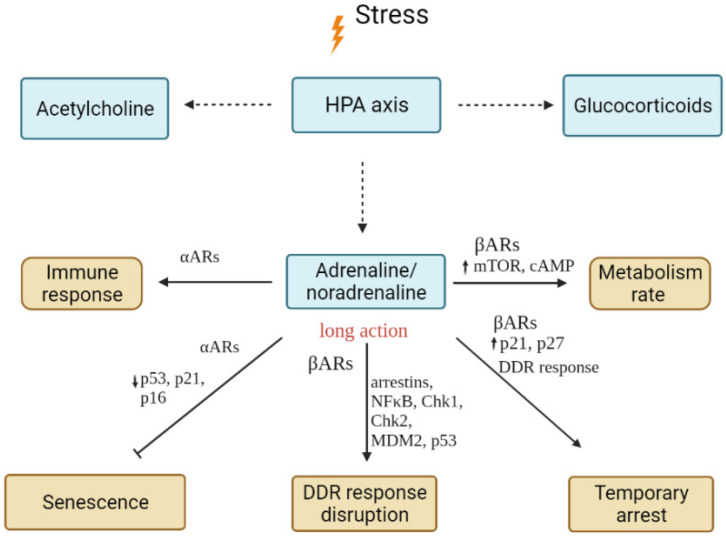
Schematic representation of effects of adrenaline and noradrenaline, sympathetic system neurotransmitters on intracellular processes and signalling pathways related to senescence, apoptosis, and cell cycle regulation. **↑**—stimulation, ↓—inhibition, αARs—α-adrenoreceptors, βARs—β-adrenoreceptors.

*Summary*: The net effect of neurotransmitters depends on the expression of receptors on specific cells. However, the effects of beta-adrenergic stimulation are apparently associated with a general activation of cell metabolism and activity, which leads to inevitable failures in cellular machinery (Figure 3). This in turn leads to the activation of protective mechanisms and temporary or permanent arrest of the cell cycle, and long-term stimulation can lead to the activation of mechanisms associated with ageing.

#### 4.2.2. Acetylcholine

Acetylcholine stimulation, in general, opposes the effects of adrenergic stimulation on the metabolic systems and signalling pathways. Activation of α7nAChR reduces ischaemia and glucose-deprivation-induced apoptosis in neurones and stimulates autophagy, which is accompanied by a decrease in the activation of the mTOR cascade and an increase in the activation of AMP-activated protein kinase (AMPK), which responds to a decrease in the balance of ATP/AMP towards AMP [112]. It should be noted that the response of AMPK to energy levels is opposite to that of mTOR, and these two kinases mutually suppress each other [113]. In addition, in astrocytes, the specific α7nAChR agonist protected cells from 1-methyl-4-phenylpyridinium (MPP+)-induced apoptosis by increasing the expression of the anti-apoptotic Bcl-2 protein, reducing the level of the apoptotic Bax protein, and activating caspase-3 via the JNK-p53 signalling pathway [114]. Activation of α7 nicotinic receptors by the specific agonist PNU-282987 blocked angiotensin II (Ang II)-induced senescence in cultured vascular epithelial cells. Such activation suppressed the activity of pro-senescent signalling pathways such as p53, acetyl-p53, p21, and p16^INK4a^ and also increased the activity of sirtuin-1 by increasing the level of NAD(+) through pathways not associated with cAMP-dependent protein kinases [115].

Cholinergic signalling influences the cell cycle. In general, nicotine, an α7 nicotinic receptor agonist, has been shown to enhance the proliferation of various cancerous and normal cell lines [116,117]. Binding of nicotine to nAChRs resulted in the formation of a complex including β-arrestin, Src, and nAChRs, which promoted Src activation. This activation stimulated the MAPK signalling pathway and resulted in binding between Raf-1 and Rb and the binding of multimeric complexes of E2F, Rb, and Raf-1 to proliferation promoters. Prolonged mitogenic signalling led to the dissociation of Rb and Raf-1 from E2F at the proliferative promoter and caused the cells to enter the S phase. An alternative pathway was associated with Ca entry via nAChRs and activation of ERK and MEKK1. Furthermore, MEKK1 activated the NF-kB pathway, leading to its entry into the S-phase [117]. However, it should be noted that agonists have a much longer binding time to the receptor than that of acetylcholine, which is rapidly eliminated by enzymes such as cholinesterase, and their effects may differ from those of acetylcholine.

Stimulation of the muscarinic acetylcholine receptors (mAChR) activated PARP1 polymerase in hippocampal cells via a Ca mobilization-dependent and an ROS-independent process [118]. PARP1 (poly(ADP-ribosyl)polymerase) is directly involved in the DDR response, participating in the poly-ADP-ribosylation of several proteins associated with the regulation of chromatin architecture and DNA metabolism and tracking DNA damage and repairing breaks in DNA [119]. mAChR activation also slowed myocardial ageing by inhibiting caspase-1 and IL-1 [120]. Activation of muscarinic receptors increased the expression of PCNA and p53 genes at the transcriptional and translational levels, expression of the Ki-67 gene at the transcriptional level, and expression of p21 and cyclin D1 at the translational level [121].

**Figure 4:**
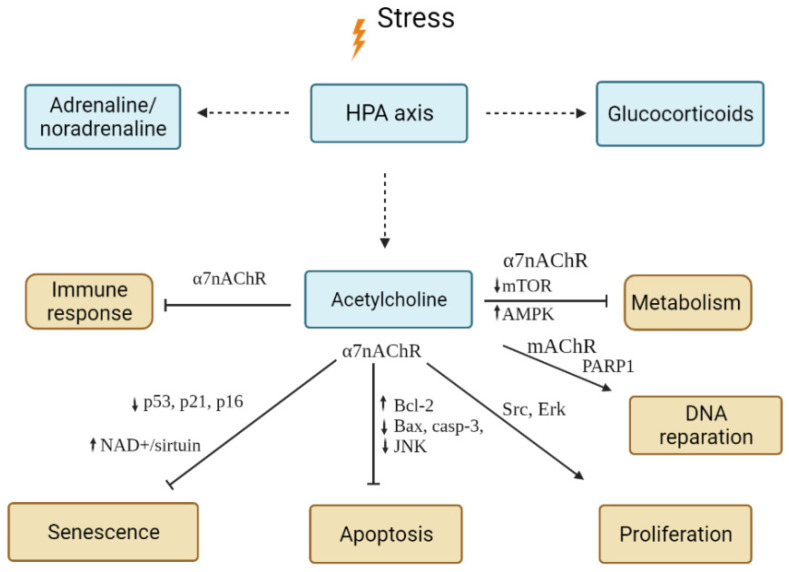
Schematic representation of effects of acetylcholine, the parasympathetic system neurotransmitter, on intracellular processes and signalling pathways related to senescence, apoptosis, and cell cycle regulation. **↑**—stimulation, ↓—inhibition, α7nAchRs—α7-nicotinic acetylcholine receptors, mAChR—muscarinic acetylcholine receptors.

*Summary:* In general, cholinergic effects lead to a decrease in the metabolic rate in activated cells and the activation of defence mechanisms associated with the elimination of DNA or organelle damage through the activation of DDR responses and the activation of autophagy (Figure 4). Such effects are generally anti-apoptotic, anti-senescent, and pro-proliferative, although the effects also depend on the receptor repertoire on specific cell types. Meanwhile, prolonged activation of nicotinic acetylcholine receptors, which was found to occur in smokers, can lead to excessive activation of pro-proliferative pathways, such as Akt, and can transform cells into cancer cells [116].

### 4.3. Alarmins

The term ‘alarmins’ refers to endogenous molecules that signal damage in cells and tissues. When combined with exogenous molecules called pathogen-associated molecular patterns (PAMPs), they form a group called damage-associated molecular patterns [122]. It is known that alarmins are released into the external environment by stressed cells, warning the surrounding tissues and the immune system about the disturbance of intracellular homeostasis, and are important regulators of inflammation. One of the most studied alarmins at the present time is the high-mobility group box 1 protein (HMGB1). This is an intranuclear protein involved in the regulation of gene transcription, DNA repair, and other processes. In addition, it was passively released from necrotic cells or actively secreted from stressed cells, inducing inflammation [123]. It was shown that large amounts of extracellular HMGB1 induced the release of pro-inflammatory cytokines, such as TNF, IL-1, and IL-6 [124]. HMGB1 release began with the relocation of the HMGB1 nuclear pool into the cytosol [125]. It has been demonstrated that the HMGB1 receptor is a receptor for advanced glycation end products (RAGE), and HMGB1 is one of the many ligands of this receptor [126]. The HMGB1-RAGE interaction did not directly activate intracellular pro-inflammatory signalling cascades. For example, macrophages expressing RAGE but not TLR4 did not produce pro-inflammatory cytokines in response to HMGB1 stimulation [127]. Recently, RAGE has been shown to transport HMGB1 and its complexes with other molecules into lysosomes through endocytosis [128]. Meanwhile, HMGB1 is involved in the destruction of the lysosomal membrane and release of the intact contents of lysosomes into the intracellular environment, which upon reaction with cytosolic receptors lead to the formation of inflammasomes [129,130,131]. In addition, the HMGB1 molecule itself, in a certain weakly oxidised state, can bind to a TLR4-receptor-like bacterial LPS and induce pro-inflammatory cascades such as the NF-κB pathway [132].

Other alarmins that are passively released from dying cells include several cytokines that are structurally similar to HMGB1, such as IL-1α, IL-33, and IL-16, and their precursors have been shown to exert certain functions in the nucleus; in this regard, they are called dual-acting cytokines [133]. Under normal conditions, the precursors are constitutively expressed in many cell types located predominantly in the nucleus. They do not require additional processing and are released during cell necrosis [134,135]. Alarmins also include compounds such as RNA, DNA, ATP, and many other substances that enter the extracellular space as a result of cell death; however, these substances are secreted passively, unlike HMGB1.

HMGB1 has been shown to form a complex in the nucleus with NAD-dependent deacetylase sirtuin-1 (SIRT1), which plays an important role in slowing senescence, as it promotes DNA repair by deacetylation of some repair enzymes, such as Ku70 [136]. SIRT1 also maintains the integrity of the genome, ensuring the appropriate level of chromatin condensation. SIRT1 normally inhibits the release of HMGB1, while activation by pro-inflammatory stimuli leads to the dissociation of HMGB1 from SIRT1 and its release. The interaction between these proteins is critical, for instance, in LPS-induced sepsis [137]. SIRT1 levels are reduced in senescent fibroblasts, pulmonary epithelial cells, endothelial cells, and macrophages [138,139,140,141], which apparently promote the release of HMGB1. Indeed, it has been shown that HMGB1 leaves the nucleus at the early stages of senescence [142].

In cells, exposure to IL-1-like substances such as HMGB1, IL-1α, IL-33, and IL-16 may induce cell cycle arrest and serve as markers of ageing. In particular, HMGB1 is an activator of p53, which increases its binding to DNA and promotes cell cycle arrest or apoptosis [142].

*Summary:* HMGB1 and other alarmins have cytokine-like effects on immune cells, causing their activation and promoting the development of an inflammatory response. Chronic exposure to alarmins, under either stressful conditions or a low-intense local inflammatory response caused by senescent cells with SASP, may spread to neighbouring cells upon attaining a threshold value, causing their cell cycle arrest and senescence. In some aspects, alarmins are similar to thymic hormones, which are explored below. 

### 4.4. Thymic Hormones

Thymus peptide hormones are interesting compounds from the perspective of inflammatory response regulation. First, they ensure maturation and proliferation of lymphocyte precursors inside the thymus, and this was previously considered their main purpose. However, this function is not their only function. Thymic hormones are secreted by the thymus into the systemic circulation and have pronounced effects on mature immune cells outside the thymus. Therefore, they are closely integrated into the HPA axis, and their concentrations fluctuate according to circadian fluctuations in corticosteroid levels. These hormones have multidirectional effects on the immune system and act as either pro-inflammatory or anti-inflammatory factors [143]. Another feature of these hormones is their great similarity with alarmins; the precursors of these peptides are ubiquitously expressed major proteins that are predominantly localized in the nucleus. Under stress conditions, these precursors are cleaved, released into the cytoplasm and/or into the extracellular environment, and serve as signals of various imbalances in intracellular processes [144]. Therefore, it is very interesting to explore how these local and systemic hormones-alarmins affect signalling pathways associated with cell cycle regulation and senescence, especially since the systemic production of these hormones decreases significantly with age due to thymic involution, although some levels persist in the elderly.

#### 4.4.1. Thymosin-α

Thymosin-α (Tα), a systemic hormone, is produced by the thymus. Its concentration is high in the embryonic phase and in neonates but falls rapidly during childhood as T-lymphocytes mature [145]. In adults, the concentration of Tα in the blood is about 0.5 ng/mL [146]. Tα is a relatively short-lived peptide; its in vitro plasma half-life is approximately 127 min [147]. Tα has shown clear interrelations with the HPA axis. In blood plasma, the concentration of Tα is inversely correlated with the circadian cycles of HPA hormones; changes in the concentration of corticosteroids affect the level of Tα, and vice versa [143]. When administered intraventricularly, Tα reduces the production of ACTH, thyrotropin, and prolactin but not the level of growth hormone [148].

The Tα precursor prothymosin-α (ProTα) binds to histones [149], histone acetyltransferase p300 [150], and CREB-binding protein [151]. The intracellular level of ProTα increased in cells during normal and pathological proliferation, resembling the effects of known oncogenes such as Ras [152]. The intracellular activity of ProTα resembled that of the HMG proteins, and their functions in the nucleus may be similar [150]. Artificially induced overexpression of ProTα leads to a p53-mediated response to cell cycle arrest [153]. In the cytoplasm, ProTα inhibits pro-apoptotic pathways [154] and promotes cell survival. In addition, by activating the Nrf2 signalling pathway, ProTα is involved in the response to oxidative stress, as Nrf2 induces a number of genes associated with antioxidant defence [155]. Increased ProTα expression in cancer cells correlates with poor prognosis [156].

In our previous review [144], we described results indicating that when cells were exposed to necrotising, but not apoptotic stimuli, there were changes in the localisation and secretion of ProTα, which then cleaved to form Tα. This secretion occurs very quickly, since it does not require additional synthesis and ProTα is already present in the cell in large amounts, comparable to the amounts of histones. Matsunada and Ueda [157] demonstrated that ProTα secretion occurs in a non-vesicular manner, with the participation of the S100A13 carrier molecule from the S100 protein family. The authors noted that this method of secretion is characteristic of necrotic stress, not non-apoptotic stress. In addition, it was noted that a necrotic stimulus (a drop in the ATP level) in the cell led to the release of almost all ProTα from the nucleus, indicating that the presence of this peptide in the extracellular environment may be one of the first signs of necrosis. This secretion can serve as a signal for the immune system. 

The immune system responds to ProTα by activation, and TLR4 stimulation is the proposed mechanism of ProTα reception [158]. Alternatively, the effects of Tα are generally directed towards modulating the activity of already activated immune cells and inducing tolerance. In addition, Tα has powerful antioxidant and anti-inflammatory effects. Detailed data have been presented in our previous review [144]. Based on these results, we hypothesised that the successive appearance of ProTα and Tα in the extracellular space reflects different stages of the response to damage, with ProTα activating certain immune functions and Tα deactivating them in a feedback loop, thereby limiting the immune response. Thus, we concluded that the ProTα/Tα system is one of the fastest lines of damage signalling, and the response to such a signal is the activation of immune cells. With further development of the immune response, ProTα is cleaved to form Tα, which has the opposite effect, at least in terms of immune activation [144].

In this regard, the effect of such a peptide on the cell cycle is of great interest. Data indicate that Tα has different effects on immune and non-immune cells. Tα predominantly stimulates the activity and proliferation of immune cells and increases their survival. Roy et al. [159] showed that Tα inhibited thymocyte apoptosis induced by the serum of tumour-bearing mice, and the mechanism was associated with the activation of protein kinase C (PKC), a decrease in the levels of pro-apoptotic proteins Bax and Bad, and an increase in the level of anti-apoptotic protein Bcl-2. In another study by the same group, it was shown that Tα had a stimulating effect on bone marrow cell proliferation and also contributed to overcoming the inhibition of myelogenesis in the development of lymphoma [160].

Alternately, in several cell lines, Tα showed antiproliferative and cytotoxic effects. In particular, Tα inhibited proliferation and induced apoptosis in leukaemic cells [161]. Guo et al. showed that Tα suppressed proliferation and induced apoptosis of breast cancer cells, and it was clearly demonstrated that the effect depended on an increase in PTEN expression, which, in turn, led to inhibition of the PI3K/Akt/mTOR signalling pathway, the activation of which is an important oncogenic mechanism [162]. Simultaneously, Tα at high concentrations had a cytotoxic effect on the A549 lung adenocarcinoma line [163]. A similar effect was observed in B16F10, H460, and HT-29 cell lines (melanoma, stomach cancer, and lung cancer) [164].

**Figure 5:**
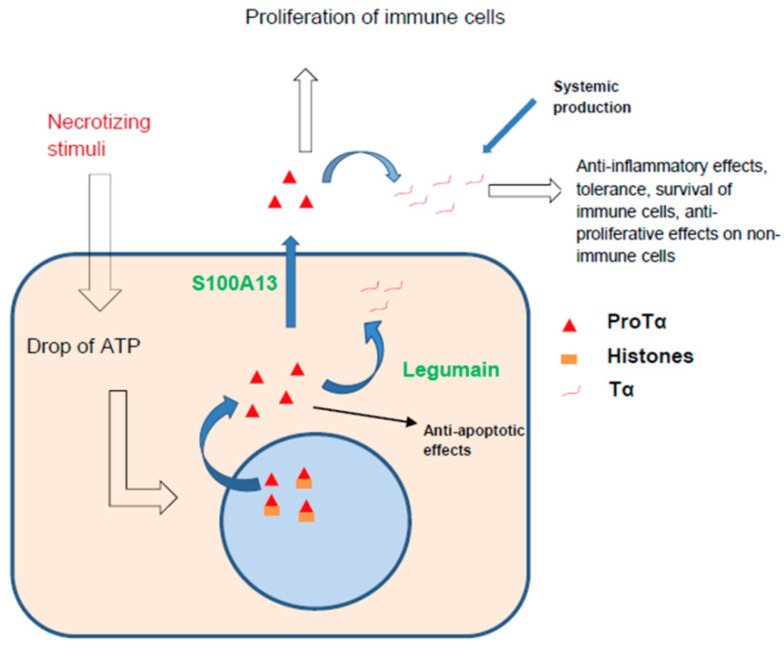
Schematic representation of intracellular and extracellular effects of prothymosin-α (ProTα) and thymosin-α (Tα). Adapted from our earlier publication [144].

*Summary:* Strong stress induces relocation of ProTα from the nucleus to the cytoplasm, which, in general, has a protective and anti-apoptotic effect, activating antioxidant systems and cell cycle arrest through a p53-dependent mechanism. When the protective capabilities of ProTα are exceeded, it is released both actively through secretion by the carrier, and passively when the cell is destroyed. Extracellularly, ProTα activates the cells of the immune system and, together with Tα, promotes their proliferation; however, other non-immune cells probably respond to Tα by cell cycle arrest mediated through PTEN. The cleavage of ProTα with the formation of Tα contributes to the inhibition of the immune response (Figure 5). 

#### 4.4.2. Thymopoietin

Like Tα, thymopoietin (Tmpo) is a short-lived peptide with a plasma half-life of 5–6 min [165]. It is produced by the thymus, and its levels in the human blood are high during youth (1 ng/mL) and decrease with age (0.25 ng/mL at the age of 50) [166]. Tmpo is integrated into the HPA axis; its administration to children results in increased levels of ACTH and cortisol [167], and it directly stimulates the production of ACTH, beta-endorphin, and beta-lipotropin in cultured pituitary cells [168]. Detailed data are presented in our previous review [143].

Tmpo has a balancing effect on immune cells. Our studies have shown that in the peritoneal macrophages and splenocytes of healthy animals, it induced the production of pro-inflammatory cytokines. However, when the cells were stimulated with bacterial LPS in a mice sepsis model, it reduced the activation of the pro-inflammatory NF-κB cascade and normalised the production of cytokines [169,170].

Tmpo, like Tα, is also a fragment of a ubiquitously expressed protein. It is an N-terminal fragment (49 amino acid residues) of an LAP2 protein (lamina-associated polypeptide-2) associated with the structural proteins of the nuclear lamina, which forms a fibrous carcase on the periphery of the nucleus, as well as with chromatin. LAP2 family proteins play a fundamental role in nuclear architecture and chromatin organisation. The membrane-associated form, LAP2β, binds to lamin B and chromosomes [171,172] and the BAF chromosomal protein [173]. It is involved in the binding of nuclear membranes to chromosomes after mitosis and in the regulation of nuclear envelope assembly [174,175]. Another form, LAP2α, is localised throughout the nucleus and binds to lamin A [176] and chromosome telomeres [177]. Suppression of LAP2 expression with long antisense RNA (LncRNA TMPO-AS1) [178] or miRNA (e.g., miR-139-5p) [179] suppresses proliferation and induces cell cycle arrest and apoptosis.

Interestingly, in Hutchinson–Gilford progeria, which is characterised by accelerated ageing, a progerin, which is a truncated form of lamin A, is produced as a result of mutation. This form was shown to have poor binding to LAP2α, and since 50% of telomeres are bound to the lamina, telomere association with LAP2α is disrupted, probably leading to telomere damage and accelerated ageing. Additionally, an artificial increase in LAP2α expression reduced the effect of progerin on proliferation, while a decrease in expression exacerbated it [180]. Interestingly, LAP2α was shown to be cleaved by caspases 3 and 6 during apoptosis, with the formation of a 50 kDa N-terminal segment (containing the Tmpo sequence) and a 28 kDa C-terminal segment. These segments were no longer associated with chromatin, and the N-terminal fragment was solubilised, while the C-terminal fragment remained associated with membrane structures [181]. It should be noted that LAP2 cleavage, specific to apoptosis and not necrosis, occurred at an early stage, while other lamina-associated proteins were not cleaved during apoptosis or were cleaved at later stages (Emerin, LBR) [182,183]. 

It should be noted that anti-tumour effects of Tmpo have long been known, but they are associated mainly with the stimulation of T-lymphocytes and the influence on their differentiation and subpopulations, not with a direct effect on tumour cells and their cell cycle. There have been limited data on how Tmpo affects cell cycle. In a model of ovarian failure syndrome, the administration of thymopentin, a pentapeptide that is an immunologically active fragment of Tmpo, was shown to have a beneficial effect on cellular ageing. This syndrome is characterised by oxidative damage to the granular cells of the ovaries, leading to ageing and apoptosis. Tmpo reduced the expression of p16 and γ-H2AX and increased the expression of Ki67, Bcl2, and Lin28 (a marker of proliferation of ovarian granulosa cells). The authors also showed that thymopentin activated the transcription factor YY2, which triggered the transcription of the Lin28 gene, which, in turn, inhibited the expression of let-7 microRNAs, affecting the cell cycle and inhibiting proliferation [184]. Recently, the YY2 transcription factor was shown to be a positive regulator of p21 expression, acting independently of p53. In addition, it is able to bind to the p53 promoter, enhancing its transcription, and therefore, YY2 can be considered an important novel cell cycle regulator [185].

Alternately, thymopentin had a different effect on actively proliferating cells. In particular, a dose-dependent inhibitory effect of thymopentin on the promyelocytic neutrophilic leukaemic cell line HL-60 was shown. Thymopentin caused the accumulation of cells in the G_1_/G_0_ phase and their differentiation into granulocytes but had no effect on PBMCs. In addition, an agonist of nicotinic acetylcholine receptors that did not affect muscarinic receptors reduced the inhibitory effect of thymopentin on the proliferation of these cells [186], which, in general, is consistent with the pro-proliferative effects of acetylcholine. In addition, thymopentin induced the maturation of bone marrow dendritic cells [187]. Thymopentin also increased the sensitivity of HL-60 cells to therapeutic doses of radiation exposure, stopping the cell cycle in the G_2_/M phase and inducing apoptosis [188].

**Figure 6:**
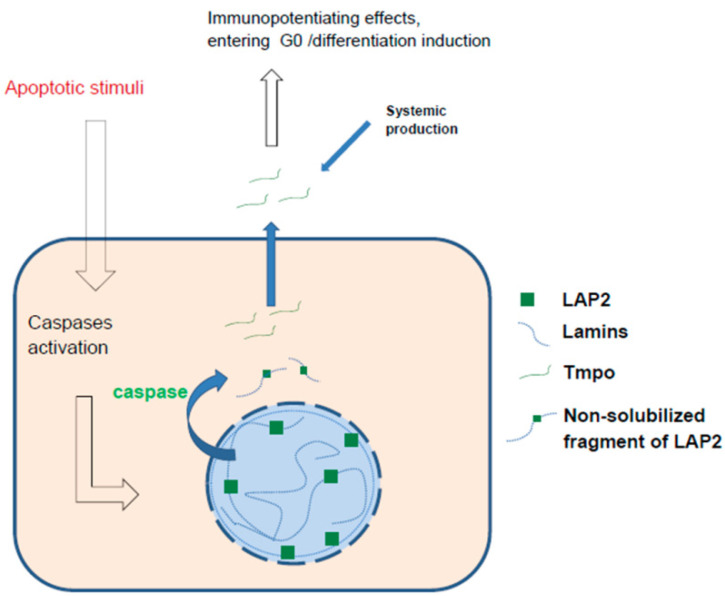
Schematic representation of intracellular and extracellular effects of lamina-associated protein 2 (LAP2) and its fragment thymopoietin (Tmpo). Adapted from our earlier publication [144].

*Summary:* Tmpo, a fragment of LAP2, plays an important role in cell division and cell cycle regulation. As a result of apoptotic but not necrotic processes, LAP2 is cleaved and solubilised, and a Tmpo-containing fragment may theoretically be released into the extracellular space (although this is not shown directly) (Figure 6). In an extracellular environment, Tmpo regulated the activity of immune cells and caused the maturation of bone marrow cells. In general, the effect of extracellular Tmpo on the surrounding cells is rather anti-proliferative, but it is not pro-senescent, i.e., it directs the cell towards the G_0_ phase and differentiation and away from the senescent pathway, that is, restoring the normal state after stress exposure. This effect may be realised through the YY2 cascade, which has not been thoroughly investigated to date. 

#### 4.4.3. Thymosin-β4

Thymosin-β4 (Tβ4) is a short-lived, acidic peptide of 43 amino acids that is present in high concentrations in plasma and increases with age, unlike other thymic hormones [189,190]. Although it has no direct effect on ACTH or corticosteroid levels when administered to the lateral ventricles, it is directly linked to the renin–angiotensin system (via the angiotensin-converting enzyme ACE, which causes degradation of its biologically active fragment, AcSDKP) and thus is also involved in the systemic control of inflammation [191]. In addition, its level in blood plasma was shown to rise sharply during exercise, which allows it to be classified as an execrine [192].

The active role of Tβ4 in wound healing has been demonstrated in many models. This peptide is present in cells in very large amounts, as it is involved in the sequestration of G-actin, a monomeric form of actin, from which actin fibres (F-actin) are generated in response to the corresponding extracellular or intracellular signals [193]. In particular, large amounts of Tβ4 contain platelets and polymorphonuclear leukocytes, which are the first to migrate into the wound site and secrete factors that attract various other cells [194]. Wound fluid contains large amounts of Tβ4 released from platelets and possibly from damaged tissue [195]. During the formation of a blood clot, Tβ4 crosslinks with fibrin, and consequently, a large amount of this peptide accumulates at the site of damage, stimulating the tissue-healing process. It was shown that Tβ4 simulated the migration of keratinocytes and endothelial cells but did not affect their proliferation [196]. Tβ4 reduced the production of key inflammatory cytokines and chemokines by inhibiting the pro-inflammatory NF-κB cascade [197,198]. Acetyl-N-Ser-Asp-Lsy-Pro (AcSDKP), a biologically active fragment of Tβ4, is produced from Tβ4 by the enzyme prolyl oligopeptidase, which is involved in the maturation of many peptide hormones [199]. This peptide stimulated the formation of capillary-like structures in vitro, increased the secretion of the active form of MMP-1, and stimulated the formation of blood vessels in vivo [200]. AcSDKP exerted an anti-inflammatory effect by inhibiting the differentiation of bone marrow stem cells into macrophages, activation and migration of macrophages, and release of pro-inflammatory cytokines by activated macrophages [201]. In plasma, AcSDKP is degraded by angiotensin-converting enzyme (ACE) [202]. It is assumed that the formation of AcSDKP leads to the inhibition of the active process of inflammation and tissue remodelling. In the early stages of healing, a large amount of Tβ4 is present in the wound; however, in subsequent stages, in response to a certain signal, endopeptidases are activated, and a large amount of AcSDKP is formed, which slows down the healing process [203]. Thus, the sequential appearance of Tβ4 and AcSDKP may reflect different stages of the wound healing process.

It may be assumed that Tβ4, as a peptide actively involved in regeneration, directly influences the processes associated with senescence and regulation of the cell cycle. Indeed, it has recently been shown that Tβ4 reduced the senescence and activation of the prosenescent factors p21, p27, cyclin D, and increased telomerase activity in a population of circulating endothelial progenitor stem cells (EPCs) upon senescence induction by serum starvation. It was concluded that this effect was associated with the activation of the Akt cascade [204]. Another study on the same cells showed that, acting through the Akt cascade, Tβ4 increased proliferation and reduced EPC apoptosis [205]. These results are also consistent with the findings that Tβ4 induced activation of the Akt pathway in medulloblastoma cells, contributing to an increase in the cellular resistance to chemotherapy with a non-elevated basal level of p53 but had the opposite effect on cells with increased p53 expression, which was caused, in particular, by oncogenic p53 mutations [206]. However, the work of Han et al. demonstrated that Tβ4 activated autophagy and protected hippocampal cells in culture from cytotoxicity caused by a prion protein, which the authors explained using a decrease in the activation of the Akt/mTOR signalling pathway, a known inhibitor of autophagy [207]. Perhaps this contradiction can be explained to some extent by the recently revealed ability of Tβ4 to induce a nonclassical autophagic pathway through HIF-1α, which is realised in response to hypoxia [208]. It has also been shown that Tβ4 is a proliferation stimulator for many types of cells, both tumour and normal. In particular, it stimulates osteoblast growth and neurite outgrowth [188], stimulates the proliferation of ovarian cancer cells [209], and has anti-apoptotic effects and increased resistance to paclitaxel in a number of cell lines [210].

**Figure 7:**
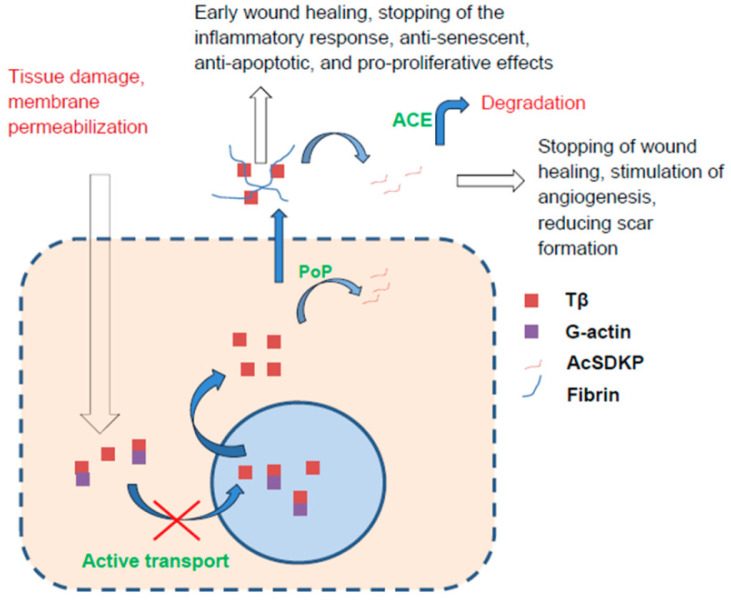
Schematic representation of intracellular and extracellular effects of thymosin-β4 (Tβ4) and its fragment AcSDKP. Adapted from our earlier publication [144].

*Summary:* Tβ4, which is released into blood plasma in large quantities during inflammation, accumulates at the sites of lesions and promotes wound healing, has a pronounced anti-inflammatory effect, and is an important pro-proliferative, anti-apoptotic, and anti-senescent modulator (Figure 7). It plays a crucial role in regeneration, but alternately, it can be a target for an anti-tumour therapy. 

#### 4.4.4. Melatonin

The HPA axis significantly depends on the central and peripheral oscillators, producing a pronounced circadian rhythm of hormone concentrations in the blood. These fluctuations directly depend on the activity of the neurones of the main pacemaker of the circadian rhythm—the suprachiasmatic nucleus. An illustration of such direct dependence is the adrenal medulla, which produces adrenaline and noradrenaline [211]. Alternately, the adrenal cortex and, consequently, the glucocorticoid rhythm have a large degree of autonomy; this rhythm is generated in the adrenal cortex and controlled mainly by ACTH and melatonin oscillations [211]. However, it should be noted that the response of the adrenal cortex to ACTH largely depends on the hormone melatonin [212,213].

Melatonin is a hormone of the pineal gland that regulates sleep/wake cycles, and its concentration attains peak levels at night. However, melatonin is present in all tissues and is produced not only by the pineal gland but also by the thymus, which is one of the main sources of melatonin in humans [214]. Melatonin acts through membrane receptors, MT1 and MT2, which are associated with G-proteins, affect cAMP levels, and mediate the main chronobiological effects of melatonin. In addition, melatonin is amphiphilic and can penetrate cells directly; therefore, the possibility of its binding to molecules inside the cell and even to nuclear receptors is being considered, although data on specific targets (calmodulin, NRH quinone oxidoreductase (NQO2), or retinoid orphan receptors (RORs)) are controversial and not fully confirmed [3]. It has been shown that in normal (non-tumour) cells, many effects of melatonin are mediated through a stimulating effect on SIRT1, which is an NAD^+^-dependent deacetylase; however, these effects depend not on the expression but on the activity of SIRT1, which requires NAD^+^, a substrate and activator of SIRT1 [215].

Melatonin directly influences immune cell activity; moreover, immune cells produce melatonin [216]. The effect of melatonin on immune cells is predominantly stimulatory and has been shown mainly in leukocytes or transformed leukocyte cells [215]. The pro-inflammatory effect of melatonin has also been shown in autoimmune diseases such as multiple sclerosis [217] and rheumatoid arthritis [218]. Pro-inflammatory effects were associated with increased production of pro-inflammatory cytokines such as IL-1, IL-2, IL-6, IL-12, TNFα, and IFNγ in various cell types, such as monocytes and T-helper type 1 (Th1) [219] and IL-17 in T-helper type 17 (Th17) cells [220]. A number of other effects of melatonin were associated with the upregulation of cytokines associated with differentiation and clonal expansion, such as macrophage colony-stimulating factor (M-CSF) and stem cell factor (SCF) in macrophages and splenocytes, transforming growth factor (TGFβ) in macrophages and dendritic cells, as well as Tα and thymulin in thymocytes [221]. However, the effects of non-immune cells are rather anti-inflammatory.

Melatonin is a highly effective antioxidant. It directly neutralises toxic reactive oxygen and nitrogen species and their metabolites [222]. The deacetylase SIRT1, which is usually classified as a type III histone deacetylase, is an important anti-inflammatory regulator responsible for many of the effects of melatonin. However, this enzyme also deacetylates several important non-histone proteins. It has been repeatedly shown that in non-tumour cells, SIRT1 expression is stimulated by melatonin, and the effects of melatonin are suppressed by SIRT1 inhibitors [215]. SIRT1 plays an important role in slowing senescence, since it promotes DNA repair by deacetylation of some repair enzymes, such as Ku70 [136], and maintains the integrity of the genome, providing the necessary level of chromatin condensation. It also forms a complex in the nucleus with the alarmin molecule HMGB1 and deacetylates it, preventing its release from cells, which is characteristic of SASP. It has also been shown that melatonin suppresses SASP [223,224,225], and the underlying mechanism may be the suppression of [poly(ADP-ribose)polymerase-1] (PARP-1)-induced expression of SASP genes [223]. Another study showed a decrease in SASP gene expression by melatonin through an increase in Nrf2 expression and a decrease in NF-kB expression [225], producing an anti-inflammatory effect.

Substrates deacetylated by SIRT1 include pathways critical for controlling cell fate, such as p53, the p65 subunit of transcription factor NF-kB, the transcription factors FoxO1 and FoxO3, Ku70, p300, Rb, E2F1, p73, and PGC-1α [226,227]. The apoptosis and growth regulator p53 is acetylated at different lysine residues by various enzymes in response to a variety of stressors, leading to increased resistance to degradation and/or increased DNA-binding capacity of p53 [226]. SIRT1 deacetylates p53, reducing its activation and attenuating the biological effects of acetylation. SIRT1 also prevents p53 translocation to the nucleus [228], which reduces the likelihood of apoptosis or senescence, but in some cases may contribute to carcinogenesis. Indeed, many studies have shown that the melatonin-induced, SIRT1-mediated decrease in senescence is accompanied by the downregulation of senescence-associated proteins p16 and p21 [229]. In addition, SIRT1 has been shown to deacetylate FoxO1 and FoxO3, both of which contribute to the inhibition of apoptosis and cell survival under oxidative stress due to SOD2 induction. Additionally, the protective effect of SIRT1 through the deacetylation of FoxO proteins under indicated conditions even prevailed over the deacetylation of p53 [230]. A large number of studies have focused on the effect of melatonin on the mTOR signalling pathway and processes associated with autophagy, but the results of these studies are contradictory and have not yet allowed for any generalisation. Melatonin has been shown to activate mTOR and suppress autophagy [231], but a different study observed the reverse [232].

*Summary:* Melatonin, acting both directly and through SIRT1, exerts an anti-inflammatory effect and shifts the balance towards increased cell survival and anti-senescent and anti-apoptotic effects. Melatonin is an important circadian oscillator, and its level fluctuates at a high amplitude in the circadian rhythm. Therefore, it can be assumed that the role of this circadian oscillator may be associated, among other things, with diurnal shifts in the metabolic balance towards energy-consuming processes such as the functioning of the immune system and reparative processes in somatic cells at night, when the energy consumption is reduced. Recent data may support this claim; studies have demonstrated that sleep disturbances can lead to a DDR response and induction of SASP [233] and impaired clearance of toxic substances, such as β-amyloid in Alzheimer’s disease [234].

**Figure 8:**
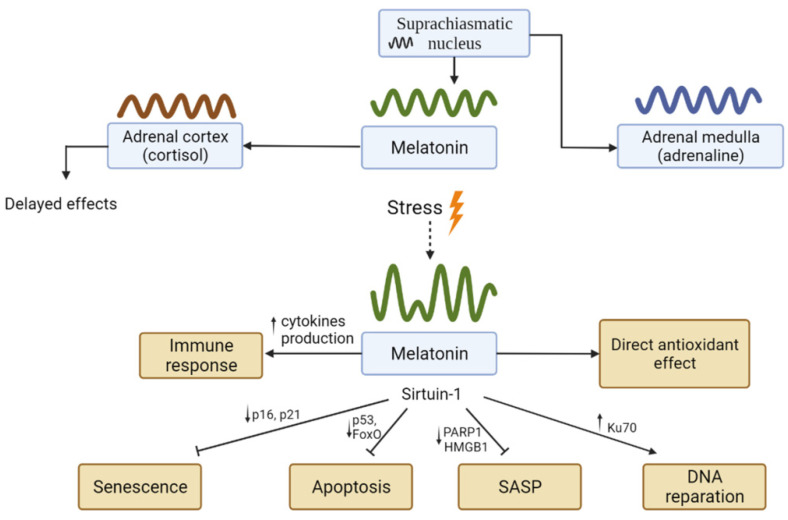
Schematic representation of effects of melatonin on intracellular processes and signalling pathways related to senescence, apoptosis, and cell cycle regulation. **↑**—stimulation, ↓—inhibition.

Another important circadian oscillator of high amplitude that needs to be mentioned in this context is the glucocorticoid rhythm. The relationship between the effects of glucocorticoids and melatonin on intracellular cascades associated with growth, senescence, and apoptosis have been questioned, given that their effects on cell cycle regulation, as described above, are generally contradictory. In this context, it should be noted that many of the effects of melatonin are either direct (e.g., antioxidant) or mediated through the activity of SIRT1, but not through its expression; therefore, they are faster than expression-mediated effects. Alternately, the glucocorticoid rhythm, firstly, follows the melatonin rhythm, and its effects, secondly, are mediated by glucocorticoid receptors, which act predominantly through transcription, or through influences on transcription factors [235] and, therefore, have some delay. Interestingly, some of the known non-genomic and faster effects of glucocorticoids are rather pro-proliferative, such as the activation of the Akt signalling pathway [236]. Thus, according to the proposed model, oscillations of glucocorticoids, whose effects are generally opposite to that of melatonin, shift the balance towards the ‘daytime’ mode, when the role of energy-consuming repair and immune processes is decreased (Figure 8). 

## 5. Conclusions

**Figure 9:**
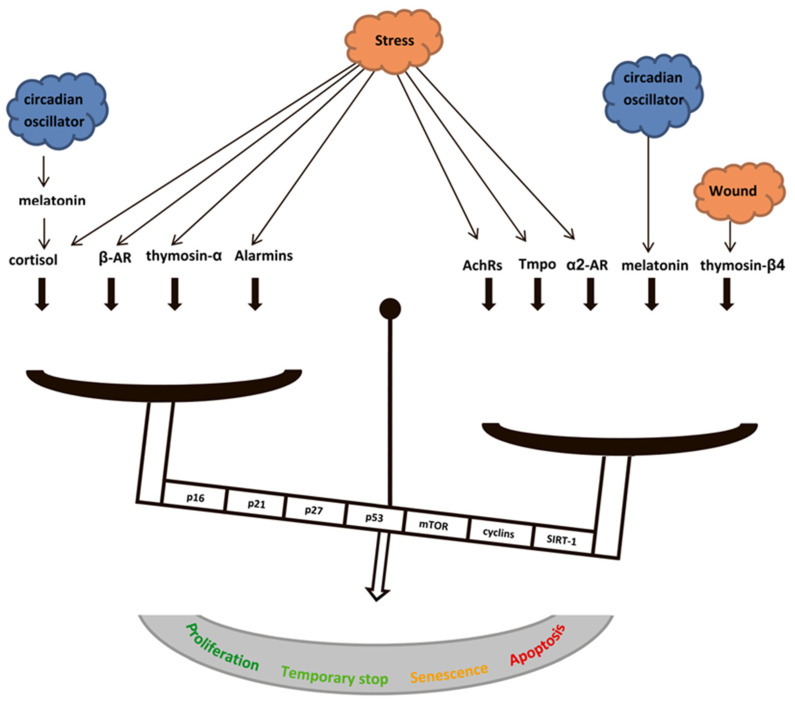
Hypothetic scheme showing the balancing effects of immune response regulators on signalling pathways that control cell cycle, senescence, and apoptosis. βAR—β-adrenoreceptors; AchRs—acetylcholine receptors; α2AR—α2-adrenoreceptors; Tmpo—thymopoietin.

The presented data have allowed us to conclude that the systemic regulators of immune response can modulate the mechanisms associated with the regulation of cell cycle, senescence, and apoptosis. Circadian oscillations in neuroendocrine systems and changes associated with the induction of stress and inflammation may shift the metabolic balance in somatic cells towards cell growth and proliferation, temporary or permanent cell cycle arrest, or apoptosis. This analysis has allowed us to postulate a multilevel system of central regulators exerting neuronal influences through neurotransmitters and endocrine influences through glucocorticoid and melatonin hormones, thymic hormones, and alarmins (Figure 9). 

This system fine-tunes the cell cycle and metabolic/catabolic processes depending on the following conditions:

1. The level of systemic stress, because it may induce activation of DDR-response after prolonged adrenalin exposure, or the cell cycle arrest after prolonged glucocorticoid exposure.
2. Stage of stress response, because it may lead to oppositely directed changes in senescence-related pathways after adrenaline/acetylcholine/cortisol release during the stress response development, release of ProTα/Tα on the different stages of cellular stresses, or Tβ4/AcSDKP release on different stages of wound healing.
3. Energy capacities of the body, because it may shift cell cycle and metabolism regulation, depending of diurnal oscillations of melatonin/cortisol concentrations. In addition, given the differently directed effects on immune and non-immune cells, this system may participate in the redistribution of body resources between an energy-consuming immune response and other functions.

These processes and levels of regulation should be considered when studying the mechanisms of ageing, proliferative processes, and programmed death at the level of the whole organism. 

## Data Availability

Not applicable.
